# Machine learning-based predictive models for the occurrence of behavioral and psychological symptoms of dementia: model development and validation

**DOI:** 10.1038/s41598-023-35194-5

**Published:** 2023-05-18

**Authors:** Eunhee Cho, Sujin Kim, Seok-Jae Heo, Jinhee Shin, Sinwoo Hwang, Eunji Kwon, SungHee Lee, SangGyun Kim, Bada Kang

**Affiliations:** 1 Mo-Im Kim Nursing Research Institute, Yonsei University College of Nursing, 50-1, Yonsei-Ro, Seodaemun-gu, Seoul, 03722 Republic of Korea; 2 Department of Nursing, Yong-In Arts and Science University, Gyeonggi-do, Korea; 3 Division of Biostatistics, Department of Biomedical Systems Informatics, Yonsei University College of Medicine, Seoul, Korea; 4 College of Nursing, Woosuk University, Jeollabuk-do, Korea; 5 Korea Armed Forces Nursing Academy, Daejeon, Korea; 6 BRFrame Inc, Seoul, Korea

**Keywords:** Signs and symptoms, Health care, Geriatrics

## Abstract

The behavioral and psychological symptoms of dementia (BPSD) are challenging aspects of dementia care. This study used machine learning models to predict the occurrence of BPSD among community-dwelling older adults with dementia. We included 187 older adults with dementia for model training and 35 older adults with dementia for external validation. Demographic and health data and premorbid personality traits were examined at the baseline, and actigraphy was utilized to monitor sleep and activity levels. A symptom diary tracked caregiver-perceived symptom triggers and the daily occurrence of 12 BPSD classified into seven subsyndromes. Several prediction models were also employed, including logistic regression, random forest, gradient boosting machine, and support vector machine. The random forest models revealed the highest area under the receiver operating characteristic curve (AUC) values for hyperactivity, euphoria/elation, and appetite and eating disorders; the gradient boosting machine models for psychotic and affective symptoms; and the support vector machine model showed the highest AUC. The gradient boosting machine model achieved the best performance in terms of average AUC scores across the seven subsyndromes. Caregiver-perceived triggers demonstrated higher feature importance values across the seven subsyndromes than other features. Our findings demonstrate the possibility of predicting BPSD using a machine learning approach.

## Introduction

The number of people living with dementia is estimated to be more than 50 million worldwide; with the increasing aging population, this number is expected to triple by 2050^1^. Behavioral and psychological symptoms of dementia (BPSD), also known as neurocognitive symptoms, are a heterogeneous group of non-cognitive symptoms and behaviors such as agitation, aggression, apathy, and depression that manifest in individuals diagnosed with dementia^2^. Almost 90% of individuals with dementia at all stages and etiologies are affected by these symptoms^3^. They are increasingly recognized as the most complex, challenging, and costly aspects of dementia care^4^, and result in decreased independence in activities of daily living and quality of life^5^, nursing home placement^6^, healthcare utilization^7^, and increased caregiver burden and depression^8^ for individuals with dementia.

The factors that have been identified as contributing to BPSD can be categorized as (1) those related to individuals with dementia, such as dementia-related neurobiological factors, unmet needs, and premorbid personality; (2) those related to caregivers, such as communication approach; and (3) environmental triggers such as lack of stimuli and environmental change^9^. They cause symptoms independently or in combination with other aspects^9^. Disturbances in circadian rhythm, including impaired sleep and inadequate physical activity, have also been identified as stressors that can trigger BPSD^10,11^. Recent empirical research has identified an association between disruption of circadian rhythms and BPSD, including depression, anxiety, agitation^12^, and sundowning-related aggressive symptoms^13^. However, given that systematic and continuous observation of sleep and activity levels is challenging to assess using traditional methods that rely on retrospective reports of caregiver observation, only a few methodologically rigorous studies have examined the influence of sleep and activity levels on such symptoms^14^.

Although a downward trajectory for the cognitive and functional decline over the course of dementia is expected, the manifestation of BPSD varies among individuals^9^. Current evidence suggests that person-centered non-pharmacological interventions that match the needs of persons with dementia and their abilities are a first-line treatment for managing BPSD. The most appropriate interventions for those with dementia should be selected individually after considering the causes and patterns of BPSD^15^. Predicting BPSD by identifying contributing factors and monitoring triggers is the first step in selecting and implementing individually customized non-pharmacological interventions to prevent and manage target symptoms^15^.

Machine learning and wearable technology have immense potential to overcome the methodological limitations of existing dementia research through the precise analysis of clinical information derived from digital devices^16^. Wearable technology such as actigraphy allows for continuous biometric monitoring, including levels of sleep and activity in everyday conditions, and for connecting them with clinical symptoms^16,17^. Machine learning facilitates the identification of underlying patterns and relationships between variables directly from data and the development of data-driven prediction models^18^. Although machine learning has been employed to develop predictive models for the incidence and detection of Alzheimer’s disease^19^, this analytic technique has rarely been applied to research on BPSD.

Hence, machine learning models are leveraged in this study to predict the occurrence of BPSD among community-dwelling older adults living with dementia based on various factors, including actigraphy-measured sleep and physical activity levels and diary-based caregiver-perceived symptom triggers.

## Methods

### Study design

This study utilized a prospective observational design with three-wave data collection to build predictive models for BPSD subsyndromes. The second wave of data collection was conducted after the first wave, with repeated measures from participants in the first wave who had agreed to participate in the second wave. A detailed description of the first and second waves of data collection is reported elsewhere^20^. In the third wave of data collection, a validation dataset was collected from new participants independently of the first and second wave data. We employed the first and second wave data for model training (i.e., the training dataset) and the third wave data for external validation (i.e., the test dataset).

We employed a standard mining methodology that comprised four steps: (1) data acquisition, (2) data preprocessing (e.g., data cleaning, class imbalance training, and dataset class optimization), (3) model learning, and (4) model evaluation^21^.

### Recruitment and data collection

The first wave of data collection was conducted between June 2018 and June 2019. Eligible older adults with dementia living at home were recruited via on-site visits from outpatient neurological clinics at two tertiary hospitals and daycare centers in Seoul and the broader Gyeonggi region in Korea. The second wave of data collection, which involved first-wave participants who agreed to continue the study, was administered between July 2019 and June 2020. For external validation, eligible participants were recruited between July 2020 and November 2020 from an outpatient neurological clinic, where the first and second waves of data collection were conducted. The inclusion criteria applied to the three-wave data collection were (1) being at least 65 years old, (2) having a diagnosis of dementia, and (3) having a score of less than 24 on the Korean version of the Mini-Mental State Examination (K-MMSE)^22^.

Eligibility screening and data collection were performed by trained research staff.

After eligibility was established, trained research staff collected demographic and health data through interviews with family caregivers and older adults with dementia. Furthermore, chart reviews and standardized scales for physical, functional, and neuropsychological assessments were administered. Following the baseline assessment, the participants wore an actigraphy device on their wrists continuously for two weeks, and primary caregivers logged BPSD in the symptom diary daily for 14 consecutive days.

### Ethical considerations

All procedures performed in this study involving human participants were in accordance with the ethical standards of the institutional or national research committee and with the Declaration of Helsinki (1964) and its later amendments or comparable ethical standards. Institutional review board approval was obtained from the Yonsei University Health System Severance Hospital (IRB 4-2018-0348, 4-2019-0314, 4-2020-0454) and Ilsan Hospital (IRB 2018-10-002-001, 2019-08-012-001). Legal representatives of all the participants provided written informed consent before enrollment after receiving a full explanation of the study procedures. The participants also provided verbal assent and written informed consent was obtained when possible.

### Features

#### Demographic and health data

At baseline, demographic and health data comprised age, sex, marital status, education level, dementia diagnosis, and neurological and psychiatric medications.

#### Cognitive and functional status

Scores (range 0–30) on the K-MMSE were used to assess cognitive functioning, with lower scores indicating greater cognitive deficits^22^. For the K-MMSE, Cronbach’s α was 0.91 in older Korean adults with dementia^23^. The severity of dementia was measured via the Korean version of the expanded Clinical Dementia Rating (CDR) scale, which assesses six functional domains—memory, orientation, judgment and problem-solving, community affairs, home and hobbies, and personal care^24^. The summed score ranged from 0 (none) to 5 (terminal dementia), and good inter-rater reliability for the overall CDR ratings was confirmed in Korean patients with dementia (kappa value range: 0.86–1.00)^25^. Functional independence was evaluated using the Korean version of Activities of Daily Living (K-ADL), which consists of seven items rated on a 3-point Likert-type scale, with higher scores indicating more severe levels of dependency^26^. The K-ADL was validated for older Korean adults with dementia, with good reliability (Cronbach’s α = 0.94)^26^.

#### Personality type

The family caregiver informant-rated premorbid personality traits of older adults with dementia were assessed using the Korean version of the Big Five Inventory (BFI-K)^27^. The BFI-K constitutes 15 items rated on a 5-point Likert-type scale that measures 5 domains of personality traits: openness, conscientiousness, neuroticism, extraversion, and agreeableness. The internal consistency of the BFI-K was good; Cronbach’s α ranged from 0.67 to 0.82^27^.

#### Actigraphy data: nighttime sleep and physical activity

Older adults with dementia were fitted with a wrist-worn actigraphy device (ActiGraph wGT3X-BT, ActiGraph Corporation, Pensacola, FL, USA), which they wore all day for 14 consecutive days. The participants were instructed to remove the device when bathing or for a few minutes as needed. Previous validity studies have demonstrated that wrist actigraphy is a reliable and suitable method for objectively measuring sleep–wake cycles in older adults with dementia^28,29^. Raw acceleration data were collected along the three axes. We used ActiLife (version 6.13.3, Pensacola, FL, US) software to export the data and process raw acceleration data to sleep and physical activity parameters using vector magnitude count in 60-s epoch data (i.e., counts per minute). The vector magnitude is calculated as the square root of the sum of the squares of acceleration for each of the three axes (x, y, z). The Cole-Kripke algorithm was applied to score a one-minute epoch as asleep or awake^30^. Moreover, the previous night’s sleep parameters were employed to predict BPSD the following day. In this study, nighttime sleep was defined as the period between 20:00 (8:00 pm) and 08:00 (8:00 am). The following nighttime sleep parameters were generated: total sleep time, wake time after sleep onset, sleep efficiency, defined as the ratio of sleep duration over the assumed sleep period (total sleep time/[total sleep time + wake time after sleep onset] × 100), number of awakenings, and mean awake length (wake time after sleep onset/number of awakenings). The following physical activity parameters were also generated: energy expenditure (calories burned) in kcal per day, metabolic equivalents per day, total time spent in moderate-to-vigorous physical activity per day, percentage of time spent in moderate-to-vigorous physical activity per day, and the number of steps per day. We employed physical activity parameters measured the same day, which reflected the physical conditions during the day when BPSD occurred.

#### Symptom diary data: BPSD and caregiver-perceived symptom triggers

A symptom diary that comprised a structured, easy-to-use checklist modeled on the Neuropsychiatric Inventory (NPI) was developed to assess the presence and severity of BPSD (i.e., delusions, hallucinations, agitation/aggression, depression/dysphoria, anxiety, elation/euphoria, apathy/indifference, disinhibition, irritability/lability, aberrant motor behaviors, sleep and nighttime behaviors, and appetite and eating disorders) daily^31^. It also included a checklist that assessed caregiver-perceived triggers of BPSD (i.e., hunger/thirst, urination/bowel movement, pain/discomfort, sleep disturbance, noise, light, temperature), interpersonal triggers (i.e., factors related to the person(s) who were present), and changes in the environment. Caregivers were also instructed to check “other causes” in the symptom diary if the perceived trigger was a factor that could not be categorized under any of the options listed in the checklist, and then, list the factors. Family caregivers were instructed to check all options that were perceived as triggers of BPSD on the same day when the symptoms had occurred. The symptom diary was designed to overcome recall bias (e.g., the NPI is based on the caregiver’s two-week retrospective rating), enable daily monitoring of the occurrence of BPSD, and link symptoms to triggers daily^32^.

Recent studies have established that clustering several individual BPSD that are highly correlated and co-occur enhances the clinical utility of the assessment of BPSD, thus allowing for a more meaningful interpretation of the study findings and increasing power by raising the number of participants who endorsed the symptom cluster rather than the individual symptoms alone^2,33,34^. Based on previous NPI factor analysis studies, we clustered certain individual symptoms into three subsyndromes: *psychotic* symptoms (hallucination and delusion), *affective* symptoms (depression, anxiety, and apathy), and *hyperactivity* symptoms (agitation/aggression, disinhibition, and irritability)^34–37^. As prior studies have demonstrated that euphoria/elation, aberrant motor behaviors, sleep and nighttime behaviors, and appetite and eating disorders do not load into any clusters^33,34,36–38^, we analyzed them as individual subsyndromes consisting of only one symptom.

### Data preprocessing

Missing actigraphy data were encountered for two main reasons: the improper wearing of the device and lack of participant compliance (e.g., constant removal of the device or not wearing the device). The number of participants with missing actigraphy data was 81/225 (36%). The mean number of days per person with missing actigraphy data was 0.9. The occurrence rates of BPSD were similar regardless of missing actigraphy data (Supplementary Table 1). Therefore, multivariate imputation was applied using chained equations^39^ to address the missing actigraphy data. Before training the models, we applied min–max normalization for continuous features. For categorical features, target encoding was employed instead of one-hot encoding. Target encoding reduces feature dimensions by converting categorical features to numerical values derived from target variables, assuming that a categorical feature is related to the outcomes^40^. There was an issue of outcome class imbalance for BPSD subsyndromes. While 26.8% of the participants exhibited affective symptoms, only 4.4% and 4.7% exhibited aberrant motor behaviors and euphoria/elation, respectively (Table 2). Researchers in various disciplines have prioritized the class imbalance problem and suggested strategies to address the issues of imbalanced data sets^41–43^. This study applied a synthetic minority oversampling technique to address the outcome class imbalance issue^44^.

### Predictive modeling

Multiple machine learning approaches were selected for this study, including logistic regression, random forest^45^, gradient boosting machine^46^, and support vector machine^47^. We investigated each of these machine learning methods with a specific learning algorithm to gauge their effectiveness, and then selected the best-performing model that could predict each subsyndrome of BPSD^18^. Using logistic regression, the most common and well-established binary classifier^48^, as the baseline model, we evaluated the degree to which the machine learning models improved performance over the baseline model.

To avoid overfitting, hyperparameter tuning through random search^49^ was implemented with five-fold cross-validation for each machine learning method. Binary cross-entropy was employed as the evaluation criterion for five-fold cross-validation. The hyperparameters for tree complexity were considered for the random forest and gradient boosting machine models. The gradient boosting machine model was iteratively trained to minimize the loss function using stochastic gradient boosting. Thus, we considered the learning rate and number of trees for the gradient boosting machine. Various kernel functions such as linear, polynomial, and radial basis kernels can be utilized for the support vector machine^50^. Linear instead of nonlinear kernels, such as the radial basis function kernel, were used in this study to prevent overfitting in small datasets and calculate feature importance. For the support vector machine models, only the regularization hyperparameter was employed to determine the optimal model. All the selected hyperparameters are described in Supplementary Table 2. Feature importance analysis was performed to investigate the contribution of a range of features in predicting the seven subsyndromes of BPSD and to sort the importance of the top 10 influential features for prediction.

### Statistical analysis

Categorical variables are summarized as the number of participants with percentages and continuous variables as means with standard deviations. Furthermore, two-sample independent *t*-tests and Fisher’s exact tests were used to compare the training and test dataset differences, respectively. The performances of the prediction models were compared and evaluated using several indices—accuracy, precision, sensitivity (recall), specificity, F1 score, and area under the receiver operating characteristic curve (AUC).

Statistical significance was set at *p* < 0.05, and all analyses were performed using R, version 3.6.3 (R Foundation for Statistical Computing, Vienna, Austria) and Python, version 3.7 (Python Software Foundation, Wilmington, USA).

## Results

### Participant characteristics

Table 1 presents the participant characteristics of both the training (*N* = 187 older adults with dementia) and test sets (*N* = 35 older adults with dementia) at baseline. The mean age of the participants was 80.4 years (*SD* 7.4) for the training dataset and 80.7 (*SD* 5.6) for the test dataset. The majority of participants were women (59% for the training dataset; 63% for the test dataset), married (62% for the training dataset; 63% for the test dataset), and with an educational level of middle school or below (57% for both the training and the test datasets). Table 1 summarizes the prevalence of BPSD among the participants.

**Table 1:** Summary statistics of study participants and prevalence of subsyndromes of behavioral and psychological symptoms of dementia (BPSD).

Participant characteristic	Training set (*n* = 187)	Test set (*n* = 35)	*p-*Value
Age (years), mean (*SD*)^a^	80.41 (7.40)	80.69 (5.56)	0.801
Gender, *n* (%)			0.851
Male	76 (40.6)	13 (37.1)	
Female	111 (59.4)	22 (62.9)	
Marital status, *n* (%)			> 0.999
Married	115 (61.5)	22 (62.9)	
Widowed or divorced	72 (38.5)	13 (37.1)	
Education, *n* (%)			> 0.999
Elementary school or below	86 (46.0)	16 (45.7)	
Middle school	20 (10.7)	4 (11.4)	
High school	48 (25.7)	9 (25.7)	
College or above	33 (17.6)	6 (17.1)	
BFI^b^, mean (*SD*)			
Openness	8.58 (2.90)	8.03 (2.77)	0.297
Conscientiousness	11.58 (2.77)	11.60 (2.77)	0.965
Neuroticism	7.56 (2.80)	7.40 (3.41)	0.771
Extraversion	8.53 (1.89)	8.83 (2.72)	0.536
Agreeableness	10.93 (2.96)	11.46 (2.37)	0.317
ADL^c^, Sum of score, mean (*SD*)	11.27 (3.88)	8.94 (2.85)	< 0.001
Companionship and mental support	1.81 (0.75)	1.51 (0.66)	0.029
Transportation and shopping	1.65 (0.77)	1.31 (0.47)	< 0.001
Preparing meals	2.03 (0.85)	1.66 (0.76)	0.016
Managing a person’s household	1.51 (0.63)	1.17 (0.38)	< 0.001
Managing medications	1.28 (0.55)	1.11 (0.32)	0.015
Communicating with others	1.32 (0.58)	1.09 (0.28)	< 0.001
Managing finances	1.65 (0.67)	1.26 (0.44)	< 0.001
MMSE^d^ score, mean (*SD*)	16.16 (6.13)	16.09 (4.71)	0.946
CDR^e^ score, *n* (%)			0.064
Questionable	26 (13.9)	12 (34.3)	
Mild	59 (31.6)	7 (20.0)	
Moderate	57 (30.5)	10 (28.6)	
Severe	35 (18.7)	6 (17.1)	
Profound	7 (3.7)	0 (0.0)	
Terminal	3 (1.6)	0 (0.0)	
Sedative (yes), *n* (%)	115 (61.5)	23 (65.7)	0.707
Dementia (yes), n (%)			
Alzheimer disease	86 (46.0)	14 (40.0)	0.581
Lewy body dementia	68 (36.6)	19 (54.3)	0.060
Vascular dementia	32 (17.2)	6 (17.1)	> 0.999
Other dementia	50 (26.9)	9 (25.7)	> 0.999
BPSD Subsyndrome^f^, *n* (%)			
Hyperactivity symptoms	88 (47.1)	19 (54.3)	0.548
Psychotic symptoms	63 (33.7)	8 (22.9)	0.288
Affective symptoms	105 (56.1)	21 (60.0)	0.813
Aberrant motor behaviors	28 (15.0)	1 (2.9)	0.093
Euphoria/elation	44 (23.5)	3 (8.6)	0.078
Appetite and eating disorders	44 (23.5)	6 (17.1)	0.542
Sleep and nighttime behaviors	68 (36.4)	13 (37.1)	1.000

^a^*SD*: standard deviation.

^b^BFI: Big Five Inventory.

^c^ADL: Activities of daily living.

^d^MMSE: Mini-Mental State Examination.

^e^CDR: Clinical Dementia Rating scale.

^f^BPSD: Behavioral and psychological symptoms of dementia.

### Summary of actigraphy and symptom diary data

Symptom diary and actigraphy data were collected over an average of 13.30 days (*SD* 2.08) per person (training set: mean [*SD*] = 13.35 [2.11]; test set: mean [*SD*] = 13.00 [1.91]).

Affective symptoms were the subsyndromes of BPSD that occurred most frequently (training set: 26.8%; test set: 17.1%), followed by hyperactivity symptoms (training set: 17.8%; test set: 12.7%). Aberrant motor behaviors, euphoria/elation, and appetite and eating disorders had lower frequency rates for both the training and test datasets. The average total sleep times per day were 7.7 h (for the training dataset) and 6.3 h (for the test dataset), and the average total nighttime sleep times per day were 5.9 h (training dataset) and 5.1 h (test dataset). The average energy expenditure was 459.9 kcal/day (training dataset) and 442.2 kcal/day (test dataset). The participants averaged 5908.7 steps (training dataset) and 5750.5 steps (test dataset) daily. Sleep disturbance (training dataset: 13.5%; test dataset: 8.4%) and interpersonal triggers (training dataset: 8.5%; test dataset: 10.8%) were the most frequently reported triggers. Table 2 provides the univariate analyses of actigraphy and symptom diary data.

**Table 2:** Descriptive statistics for actigraphy and symptom diary.

Characteristics	Training set (*n* = 2497 days)	Test set (*n* = 455 days)	*p-*Value
Sleep, mean (*SD*)^a^			
TST^b^ (min)	464.54 (260.94)	379.51 (253.26)	< 0.001
WASO^c^ (min)	54.05 (42.18)	27.90 (26.06)	< 0.001
Efficiency (min)	0.89 (0.08)	0.93 (0.06)	< 0.001
NoA^d^ (min)	19.39 (13.96)	11.69 (9.81)	< 0.001
MAL^e^ (min)	2.72 (1.10)	2.20 (1.05)	< 0.001
TST at night (min)	353.68 (171.60)	308.58 (183.47)	< 0.001
WASO at night (min)	41.55 (32.38)	22.44 (21.46)	< 0.001
Efficiency at night (min)	0.88 (0.08)	0.93 (0.05)	< 0.001
NoA at night (min)	15.32 (10.50)	9.76 (8.28)	< 0.001
MAL at night (min)	2.60 (1.15)	2.12 (1.03)	< 0.001
Activity, mean (*SD*)			
Kcals/day	459.92 (338.66)	442.18 (298.39)	0.254
METs^f^/day	1.15 (0.13)	1.14 (0.11)	0.186
MVPA^g^/day	79.70 (73.97)	75.80 (67.00)	0.261
% of MVPA/day	5.70 (5.19)	5.40 (4.71)	0.217
Steps/day	5908.73 (4501.06)	5750.51 (4151.32)	0.461
BPSD^h^ subsyndrome, *n* (%)			
Hyperactivity symptoms	444 (17.8)	58 (12.7)	0.008
Psychotic symptoms	318 (12.7)	31 (6.8)	< 0.001
Affective symptoms	668 (26.8)	78 (17.1)	< 0.001
Aberrant motor behaviors	111 (4.4)	1 (0.2)	< 0.001
Euphoria/elation	117 (4.7)	4 (0.9)	< 0.001
Appetite and eating disorders	209 (8.4)	11 (2.4)	< 0.001
Sleep and nighttime behaviors	309 (12.4)	42 (9.2)	0.059
BPSD triggers, n (%)			
Hunger/thirst	179 (7.2)	16 (3.5)	0.003
Urination/bowel movement	264 (10.6)	8 (1.8)	< 0.001
Pain/discomfort	221 (8.9)	43 (9.5)	0.656
Sleep disturbance	337 (13.5)	38 (8.4)	0.002
Noise	93 (3.7)	6 (1.3)	0.007
Light	84 (3.4)	5 (1.1)	0.007
Temperature	126 (5.0)	12 (2.6)	0.022
Interpersonal triggers	212 (8.5)	49 (10.8)	0.126
Environmental change	120 (4.8)	7 (1.5)	< 0.001
Other causes^i^	269 (10.8)	15 (3.3)	< 0.001

^a^*SD*: Standard deviation.

^b^TST: Total sleep time.

^c^WASO: Wake after sleep onset.

^d^NoA: Number of awakenings.

^e^MAL: Mean awake length.

^f^METs: Metabolic equivalents.

^g^MVPA: Moderate-to-vigorous physical activity.

^h^BPSD: Behavioral and psychological symptoms of dementia.

^i^Other causes: Any other caregiver-perceived BPSD trigger that could not be categorized as one of the options provided in the symptom diary (e.g., medical treatment, hospital visits, and nightmare).

### Performance comparison for prediction models

Table 3 presents the prediction performance of all prediction models based on the training dataset with five-fold cross-validation. Gradient boosting machine models showed higher AUC values compared to other prediction models for predicting hyperactivity (0.706), affective symptoms (0.747), and appetite and eating disorders. (0.816). While the support vector machine model demonstrated the highest AUC value (0.706) for psychotic symptoms, the random forest model exhibited the highest AUC value (0.942) for sleep and nighttime behavior. The logistic regression models denoted the highest AUC values for aberrant motor behaviors (0.822) and euphoria/elation (0.696).

**Table 3:** Performance comparison of the prediction models for subsyndromes of behavioral and psychological symptoms of dementia (BPSD) for the training dataset with five-fold cross-validation.

Subsyndrome	Model	AUC^a^	Accuracy	Precision	Sensitivity	Specificity	F1 Score
Hyperactivity symptoms	LR^b^	0.682	0.652	0.589	0.731	0.459	0.591
	RF^c^	0.682	0.680	0.524	0.912	0.108	0.483
	GBM^d^	0.706	0.695	0.590	0.890	0.216	0.548
	SVM^e^	0.689	0.652	0.594	0.720	0.486	0.597
Psychotic symptoms	LR	0.682	0.809	0.556	0.889	0.226	0.557
	RF	0.692	0.875	0.439	0.996	0.000	0.467
	GBM	0.631	0.762	0.478	0.853	0.097	0.476
	SVM	0.706	0.824	0.561	0.911	0.194	0.556
Affective symptoms	LR	0.745	0.719	0.731	0.876	0.514	0.696
	RF	0.728	0.668	0.708	0.924	0.333	0.612
	GBM	0.747	0.691	0.708	0.883	0.441	0.659
	SVM	0.737	0.730	0.739	0.869	0.550	0.712
Aberrant motor behaviors	LR	0.822	0.832	0.546	0.846	0.500	0.547
	RF	0.534	0.961	0.480	1.000	0.000	0.490
	GBM	0.609	0.961	0.480	1.000	0.000	0.490
	SVM	0.679	0.816	0.492	0.846	0.100	0.470
Euphoria/ elation	LR	0.696	0.785	0.544	0.808	0.438	0.539
	RF	0.641	0.918	0.541	0.975	0.063	0.522
	GBM	0.627	0.910	0.589	0.958	0.188	0.580
	SVM	0.632	0.816	0.546	0.846	0.375	0.550
Appetite and eating disorders	LR	0.619	0.883	0.511	0.893	0.250	0.500
	RF	0.709	0.980	0.492	0.996	0.000	0.495
	GBM	0.816	0.973	0.492	0.988	0.000	0.493
	SVM	0.629	0.895	0.514	0.905	0.250	0.507
Sleep and nighttime behaviors	LR	0.889	0.777	0.663	0.776	0.784	0.680
	RF	0.942	0.906	0.895	0.991	0.405	0.752
	GBM	0.895	0.918	0.869	0.977	0.568	0.810
	SVM	0.888	0.770	0.663	0.763	0.811	0.677

^a^AUC: Area under the receiver operating characteristic curve.

^b^LR: Logistic regression.

^c^RF: Random Forest.

^d^GBM: Gradient boosting machine.

^e^SVM: Support vector machine.

Table 4 presents the prediction performance of all prediction models based on the test dataset, and Supplementary Fig. 1 illustrates the receiver operating characteristics and precision-recall curves for the test dataset. Compared with the logistic regression models, the machine learning models revealed better performance for all seven subsyndromes. Specifically, the random forest and gradient boosting machine models performed better than the logistic regression and support vector machine models for most subsyndromes. The random forest models exhibited higher AUC values than the other prediction models for predicting hyperactivity (0.835), euphoria/elation (0.968), and appetite and eating disorders (0.888). The gradient boosting machine models presented higher AUC values than the other prediction models for predicting psychotic symptoms (0.801), affective symptoms (0.936), and aberrant motor behaviors (0.498). Moreover, the support vector machine model showed the highest AUC value (0.929) for sleep and nighttime behavior.

**Table 4:** Performance comparison of the prediction models for subsyndromes of behavioral and psychological symptoms of dementia (BPSD) for the test dataset.

Subsyndrome	Model	AUC^a^	Accuracy	Precision	Sensitivity	Specificity	F1 Score
Hyperactivity symptoms	LR^b^	0.788	0.869	0.707	0.946	0.365	0.675
	RF^c^	0.835	0.889	0.806	0.984	0.261	0.660
	GBM^d^	0.820	0.867	0.698	0.950	0.322	0.657
	SVM^e^	0.828	0.854	0.681	0.917	0.443	0.681
Psychotic symptoms	LR	0.770	0.821	0.580	0.844	0.516	0.594
	RF	0.827	0.959	0.979	1.000	0.419	0.785
	GBM	0.801	0.936	0.774	0.985	0.290	0.679
	SVM	0.786	0.823	0.591	0.842	0.581	0.609
Affective symptoms	LR	0.866	0.814	0.697	0.856	0.619	0.713
	RF	0.927	0.869	0.776	0.918	0.645	0.779
	GBM	0.936	0.885	0.801	0.920	0.723	0.810
	SVM	0.888	0.844	0.759	0.824	0.935	0.789
Aberrant motor behaviors	LR	0.128	0.966	0.499	0.968	0.000	0.491
	RF	0.339	0.998	0.499	1.000	0.000	0.499
	GBM	0.498	0.998	0.499	1.000	0.000	0.499
	SVM	0.106	0.952	0.499	0.954	0.000	0.488
Euphoria/ elation	LR	0.765	0.877	0.512	0.880	0.500	0.494
	RF	0.968	0.991	0.497	0.998	0.000	0.498
	GBM	0.955	0.989	0.497	0.995	0.000	0.497
	SVM	0.858	0.874	0.517	0.875	0.667	0.500
Appetite and eating disorders	LR	0.712	0.896	0.549	0.907	0.455	0.562
	RF	0.888	0.975	0.487	1.000	0.000	0.494
	GBM	0.862	0.968	0.632	0.988	0.182	0.603
	SVM	0.740	0.865	0.526	0.878	0.364	0.523
Sleep and nighttime behaviors	LR	0.911	0.925	0.776	0.951	0.679	0.794
	RF	0.912	0.933	0.903	0.995	0.333	0.723
	GBM	0.900	0.938	0.848	0.982	0.506	0.785
	SVM	0.929	0.935	0.800	0.956	0.728	0.819

^a^AUC: Area under the receiver operating characteristic curve.

^b^LR: Logistic regression.

^c^RF: Random Forest.

^d^GBM: Gradient boosting machine.

^e^SVM: Support vector machine.

The gradient boosting machine model achieved the best performance in terms of the average AUC scores across the seven subsyndromes for both the training and test datasets. Tables 3 and 4 present the findings for the other performance indices.

### Feature importance

**Figure 1:**
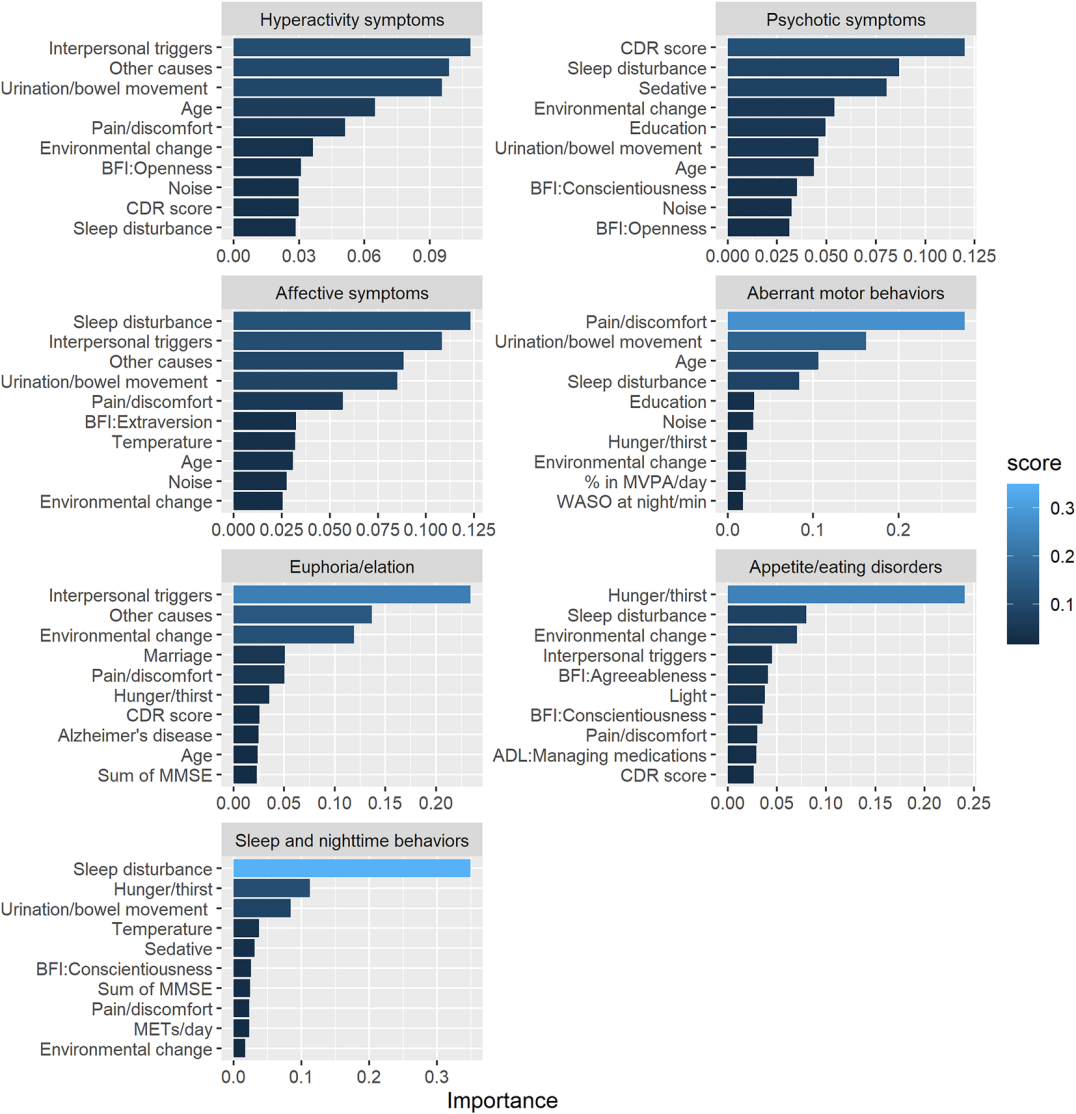
Importance of the top 10 features calculated by the gradient boosting machine for each subsyndrome of the behavioral and psychological symptoms of dementia (BPSD). “Other causes” indicates any other caregiver-perceived BPSD trigger that cannot be categorized as one of the options provided in the symptom diary (e.g., medical treatment, hospital visits, and nightmare). *ADL* activities of daily living; *BFI* Big Five Inventory; *CDR* Clinical Dementia Rating scale; *MMSE* Mini-Mental State Examination; *WASO* wake after sleep onset; *MVPA* moderate-to-vigorous physical activity; *METs* metabolic equivalents.

The top 10 most significant features of the gradient boosting machine models, which achieved the best performance in terms of average AUC scores across the seven subsyndromes, were ranked using the permutation feature importance method. Figure 1 illustrates the relative importance of the predictors included in the seven subsyndromes. Caregiver-perceived triggers reported in the symptom diary, including interpersonal triggers, sleep disturbance, urination/bowel movement, and pain/discomfort, exhibited higher feature importance values than the other features across the seven subsyndromes. The CDR score was the most influential feature for psychotic symptoms and ranked in the top 10 for hyperactivity, euphoria/elation, and appetite and eating disorders. The features for sleep and activity levels, such as waking after sleep onset at night and percentage of moderate-to-vigorous physical activity per day, were in the top ranks of feature importance for aberrant motor behaviors and sleep and nighttime behaviors. Except for two symptoms (aberrant motor behaviors and euphoria/elation), premorbid personality types were among the top 10 influential features.

## Discussion

This study constructed predictive models for seven subsyndromes of BPSD, with overall good prediction accuracy, through a supervised machine learning technique based on actigraphy measures of sleep and activity levels, symptom diary entries of caregiver-perceived symptom triggers, standardized measures of cognitive and functional status and premorbid personality type, and a medical chart review. Through the feature importance approach, we also identified the relative importance of predicting each subsyndrome. The machine learning algorithms developed and validated in our study will inform timely and appropriate preventative approaches that identify individuals at high risk for specific subsyndromes of BPSD, and thus, provide customized interventions that address the underlying causes and triggers of the target symptoms.

Although the predictive machine learning models varied among the subsyndromes with regard to different evaluation metrics, our findings demonstrated the feasibility and usefulness of the machine learning approach for predicting BPSD by accounting for multifaceted factors. Although several studies have applied machine learning algorithms with a focus on predicting the future onset of or detecting undiagnosed dementia^19,51^, few have used machine learning models to predict the various symptoms observed in older adults with dementia. A recent study employed the deep learning approach to forecast agitation episodes based on environmental stimuli data, including ambient noise level, room temperature, and relative humidity, that was collected up to 30 min before the occurrence of an actual episode^52^. While it demonstrated the feasibility and efficacy of deep learning models in predicting agitation episodes (accuracy: 98.6%; sensitivity: 84.8%), the models developed in our study were more comprehensive and practical because they examined diverse BPSD, which often co-occurs in real care settings. Besides, a machine learning approach can account for a range of multifaceted contributing factors, including actigraphy data and symptom diary data measured over time, which might not have been feasible in conventional statistical modeling owing to highly complex relationships among the variables. In recent years, big healthcare data have emerged along with advances in assistive technologies, including wearable and mobile technologies in healthcare for older adults^53^, which has increased the number of candidate features^54^. Machine learning is a promising tool in predicting BPSD arising from interactions among a web of personal, interpersonal, and environmental factors captured by various digital health technologies^54^. Moreover, machine learning algorithms that predict BPSD subsyndromes can be incorporated into a mobile app to support healthcare providers’ decision-making in selecting individually customized non-pharmacological interventions that address the underlying causes of the target symptoms^54^.

Our outcomes concerning feature importance suggested that not all features contributed consistently to the prediction of each subsyndrome. Caregiver-perceived symptom triggers, including interpersonal triggers, sleep disturbance, and pain/discomfort, were consistently ranked within the five most influential features across the seven subsyndromes. The features for sleep and activity levels, such as waking after sleep onset at night and percentage of moderate-to-vigorous physical activity per day, ranked highly in terms of feature importance for aberrant motor behaviors and sleep and nighttime behaviors. Age, severity of dementia, cognitive function, education level, and premorbid personality type were also ranked among the 10 most influential features. While activity data were relatively less influential than other features, metabolic equivalents per day were ranked in the top 10 important features for sleep and nighttime behaviors, as was wake after sleep onset at night and the percentage of moderate-to-vigorous physical activity per day for aberrant motor behaviors. The clinical implications of the results are that healthcare providers and caregivers need to consider BPSD as heterogeneous and having different important predictors. Hence, effective interventions to prevent and manage target predictors and symptoms should be employed.

While caregiver-perceived symptom triggers evaluated by the symptom diary were among the most influential features, activity data were less influential than expected for most subsyndromes in our study, except for aberrant motor behaviors. This notion is somewhat consistent with Valembois et al.’s observational study^55^, which investigated the association between motor activity and sleep duration measured by actigraphy with different types of BPSD. They found a significant increase in motor activity among those with aberrant motor behavior between 21:00 (9:00 pm) and midnight. They explained the phenomenon in terms of the sundown syndrome, referring to an increase in BPSD from late afternoon to night; otherwise, no relationship between sleep duration or night awakening episodes and any type of BPSD was found^55^. Another study utilized the machine learning approach to predict mobility, cognitive, and depressive symptoms related to Alzheimer’s disease from activity-aware smart home behavior data (e.g., activities of daily living, sleep, outings, and global routines)^56^. While statistically significant predictors were observed for mobility and cognitive symptoms, depression measured by the Geriatric Depression Scale (GDS) was weakly correlated with the global set of smart home behavioral data^56^. This outcome somewhat aligns with our finding that affective symptoms were predictable using machine learning models, but its classification (occurrence vs. absence) was mostly not influenced by actigraphy data. Additionally, the inclusion of a variety of predictors in the machine learning model might have decreased the importance of features derived from actigraphy data in our study. For clinical implications, a comprehensive monitoring system that utilizes a symptom diary written by direct caregivers, in addition to wearable sensor technologies, is required for accurate prediction.

A strength of our study is the inclusion of a variety of input data, objectively measured by actigraphy, and caregiver-perceived symptom triggers assessed using a symptom diary to develop the most powerful predictive machine learning models. Furthermore, external validation was performed using an independent test dataset to prevent overestimation of the results^57,58^. Consistent results from the training and test datasets that differed substantially in terms of severity of functional impairment, characteristics of sleep and activity levels, caregiver-perceived symptom triggers, and frequency of occurrence of symptoms can be regarded as confirmation of generalizability^58^. Finally, the symptom diary, from which the most influential features were derived, was easy to measure daily in real life. The feature importance results will facilitate the development of a digital diary that tracks BPSD using devices such as smartphones and tablets. A digital diary enables caregivers to log the symptom manifestation and circumstances, including diverse triggers, in real time, and the accumulated data can be analyzed to provide an individualized approach to symptom management.

Our study had certain limitations. First, the symptom diary was written daily, and caregivers checked one or more types of symptoms that were observed and one or more factors that were perceived as triggers for the symptoms each day. Hence, we could not disentangle which caregiver-perceived trigger contributed to which type of individual symptom observed that day. Future research needs to investigate the link between caregiver-perceived triggers and specific symptoms on an episodic rather than a daily basis. Episodic prediction algorithms can provide more precise and accurate information regarding specific symptoms. Second, the prediction performances for certain subsyndromes were poor, particularly in the case of aberrant motor behaviors (AUC = 0.498 in the gradient boosting machine model). The findings regarding the prediction performance need to be interpreted cautiously considering the small sample size of the test dataset. The frequency of occurrence of certain symptoms, particularly aberrant motor behaviors and euphoria/elation, was very low (e.g., 2/871 days of aberrant motor behavior). The overall small sample size of the test dataset might lead to lower power for pattern recognition^59^. Future replication studies with larger sample sizes are needed to obtain more accurate predictions for the subsyndromes that were less observed in our study and to prevent model overfitting and biased machine learning performance^59^. As it was challenging to collect accurate data on comorbid conditions for older adults who were recruited from other outpatient neurological clinics (i.e., daycare centers), we did not account for any comorbidities that might have affected their BPSD. Additionally, caregivers’ daily written reports were used rather than direct observation of BPSD, which could result in an observer and recall bias. While dementia care has relied on caregivers’ subjective assessment reports in most cases^4^, future studies need to employ technology such as wearable sensors, non-wearable motion sensors, or assistive and smart home technologies to objectively monitor BPSD in real time, thus revolutionizing precision dementia care^60^.

## Conclusions

This study developed and validated prediction models for BPSD subsyndromes using a machine learning approach. Overall, four classification models were trained to identify the optimal prediction model for each subsyndrome. To our knowledge, this study is the first to employ machine learning to predict BPSD using a wide variety of data, including actigraphy data. It suggests that machine learning-based prediction models can classify the manifestations of subsyndromes of BPSD. This study also identified influential predictors for specific subsyndromes that can be employed to prevent and manage target symptoms. Based on the outcomes of this study, algorithms that predict BPSD can be clinically applied to monitor and predict BPSD subsyndromes. Delivery of person-centered dementia care can be achieved through the early prediction of target subsyndromes and the provision of individually tailored non-pharmacological interventions that address the underlying causes of BPSD. Machine learning algorithms can also be embedded into smartphone applications to increase their clinical utility. Accordingly, this study is the first step toward personalized care for BPSD management using digital health technologies.

## Supplementary Information

Supplementary Information.

## Data Availability

The datasets generated and/or analysed during the current study are not publicly available due to the data security requirements of the hospital but are available from the corresponding author on reasonable request.
