# Supplementary material for: Machine learning-based predictive models for the occurrence of behavioral and psychological symptoms of dementia: model development and validation
Source: Sci Rep. 2023 May 18;13:8073. doi: 10.1038/s41598-023-35194-5 (PMC10195861; doi:10.1038/s41598-023-35194-5)
Supplement: Supplementary file 1 — Supplementary Information. [file 41598_2023_35194_MOESM1_ESM.docx]

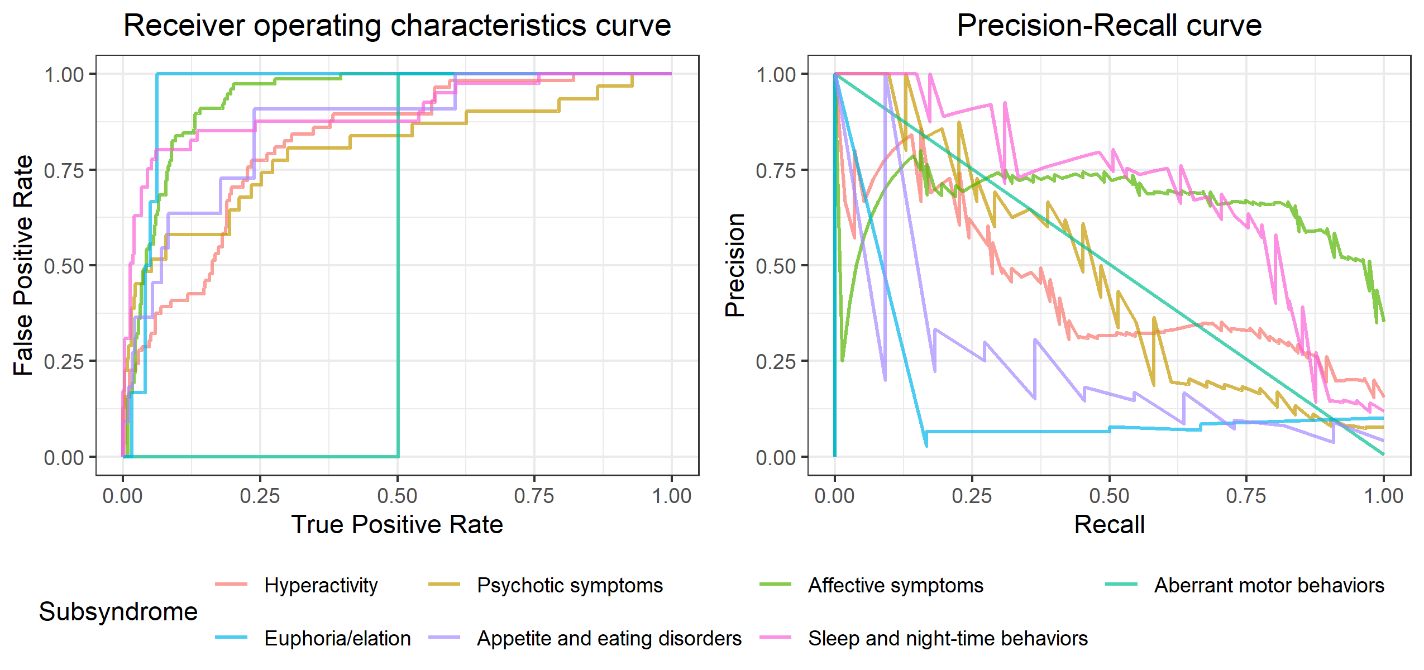


**Supplementary Figure 1.** Performance of prediction models for subsyndromes of behavioral and psychological symptoms of dementia for the test dataset. ROC: Receiver operating characteristic.

**Supplementary Table 1**. Comparison of occurrence rates of subsyndromes of behavioral and psychological symptoms of dementia depending on the occurrence of missing actigraphy data.

| Missing Actigraphy Data | | | |
| --- | --- | --- | --- |
| ^a^BPSD | Yes (n=77) | No (n=145) | Total (n=222) |
| Hyperactivity symptoms | 188 (22.6) | 314 (20.0) | 502 (20.9) |
| Psychotic symptoms | 114 (13.7) | 235 (15.0) | 349 (14.5) |
| Affective symptoms | 242 (29.1) | 504 (32.1) | 746 (31.1) |
| Aberrant motor behaviors | 31 (3.7) | 81 (5.2) | 112 (4.7) |
| Euphoria/elation | 45 (5.4) | 76 (4.8) | 121 (5.0) |
| Appetite and eating disorders | 95 (11.4) | 125 (8.0) | 220 (9.2) |
| Sleep and nighttime behaviors | 116 (14.0) | 235 (15.0) | 351 (14.6) |

^a^BPSD: Behavioral and psychological symptoms of dementia.

| Subsyndromes of behavioral and psychological symptoms of dementia | RF^a^ | | GBM^b^ | | | | SVM^c^ |
| --- | --- | --- | --- | --- | --- | --- | --- |
|  | min samples leaf | min samples split | learning rate | max depth | min samples leaf | ratio of subsample | regularization parameter |
| Hyperactivity symptoms | 1 | 2 | 0.22 | 7 | 7 | 0.90 | 7.15 |
| Psychotic symptoms | 1 | 3 | 0.28 | 7 | 3 | 0.78 | 10.06 |
| Affective symptoms | 1 | 3 | 0.07 | 6 | 4 | 0.98 | 9.41 |
| Aberrant motor behaviors | 1 | 3 | 0.11 | 5 | 3 | 0.60 | 9.55 |
| Euphoria/elation | 1 | 3 | 0.16 | 7 | 7 | 0.54 | 7.89 |
| Appetite and eating disorders | 1 | 4 | 0.19 | 7 | 7 | 0.51 | 10.06 |
| Sleep and nighttime behaviors | 1 | 2 | 0.23 | 7 | 4 | 0.88 | 9.90 |

**Supplementary Table 2.** Summary of selected hyperparameters for machine learning models.

^a^RF: random forest.

^b^GBM: gradient boosting machine.

^c^SVM: support vector machine.
